# The curse of idiopathic

**DOI:** 10.1007/s10545-017-0101-z

**Published:** 2017-10-27

**Authors:** Kevin J. O’Brien, William A. Gahl, Bernadette R. Gochuico

**Affiliations:** 1 Office of the Clinical Director, National Human Genome Research Institute, National Institutes of Health, Bethesda, MD USA; 2 Medical Genetics Branch, National Human Genome Research Institute, National Institutes of Health, 10 Center Drive, MSC 1851, Bethesda, MD 20892-1851 USA

The anti-fibrotic drug pirfenidone has proven efficacy in slowing lung function decline in idiopathic pulmonary fibrosis (IPF). The registration studies were designed to include patients with IPF and to exclude patients with pulmonary fibrosis associated with other disorders. Although the patients investigated in these studies comprised a pathogenically heterogeneous cohort, Food and Drug Administration (FDA) approval of pirfenidone applies only to patients deemed to have *idiopathic* causes for their pulmonary fibrosis. Consequently, use of pirfenidone for non-idiopathic causes of pulmonary fibrosis is considered off-label, subject to prescribing liabilities and insurance denial.

The irony is that IPF patients do have a cause for their disease; we just do not know what it is. Although IPF patients may benefit from pirfenidone, as soon as the cause of their disorder is identified, they no longer have IPF and become ineligible for receiving pirfenidone. Worse yet, patients with certain types of pulmonary fibrosis have unknown mechanisms for their lung disease, but have a name attached to their disorder (e.g., Hermansky-Pudlak syndrome [HPS], lysinuric protein intolerance, Niemann–Pick disease type B, Gaucher disease), rendering their diseases not idiopathic.

HPS is a rare autosomal recessive disorder characterized by progressive pulmonary fibrosis in adults with the most common genetic type, HPS-1. The etiology of pulmonary fibrosis remains unknown in patients with HPS or inborn errors of metabolism, but these patients are not considered to have IPF simply because a name has been attached to their lung disease. Consequently, they have difficulty obtaining pirfenidone, despite two trials that were either inconclusive or suggestive of efficacy in HPS. Thus, diagnosing HPS or inborn errors of metabolism in a patient with pulmonary fibrosis, while a diagnostic success, can prove fatal for the patient; instead of personalized medicine bringing precision therapy, it brings a death sentence.

Although action must be taken, there really is no culpability. Physicians are understandably reluctant to prescribe off-label medications; off-label use cannot be promoted; and the FDA can only approve an indication for which it has received data.

But a word—one single word—now defines life and death for individuals with pulmonary fibrosis. Not for logic or reason or ethics or politics, but by chance alone, the moniker “idiopathic” was attached to a large group of etiologies of pulmonary fibrosis and excluded subpopulations with diagnoses also associated with enigmatic fibrotic lung disease. What if the patients studied in registration trials were said to have simply pulmonary fibrosis, and not IPF? Then all patients with pulmonary fibrosis, including those with HPS or inborn errors of metabolism, would be eligible for pirfenidone.

Potential solutions to this conundrum require consolidated efforts. One by one, studies to treat each subpopulation with non-idiopathic pulmonary fibrosis could be performed, and applications could be submitted to the FDA for approval or denial. Meanwhile, the clock is ticking for many individuals with non-idiopathic pulmonary fibrosis, who, cursed by a single word, are ineligible for anti-fibrotic drugs, and hope for an opportunity to be treated for their incurable disease.

